# Thrombectomy for Acute Ischemic Stroke With a New Device-Skyflow: Study Protocol for a Prospective, Multicenter, Stratified Randomized, Single-Blinded, Parallel, Positive Controlled, Non-inferiority Clinical Trial

**DOI:** 10.3389/fneur.2021.645431

**Published:** 2021-04-30

**Authors:** Huan Liu, Zhaoshuo Li, Liangfu Zhu, Tengfei Zhou, Qiaowei Wu, Yanyan He, Xintong Song, Yingkun He, Tianxiao Li

**Affiliations:** ^1^Department of Interventional Neuroradiology, Zhengzhou University People's Hospital, Henan University People's Hospital, Henan Provincial People's Hospital, Henan Provincial Neurointerventional Engineering Research Center, Henan International Joint Laboratory of Cerebrovascular Disease and Henan Engineering Research Center of Cerebrovascular Intervention, Zhengzhou, China; ^2^Norman Bethune College of Medicine, Jilin University, Changchun, China

**Keywords:** skyflow, thrombectomy, acute ischemic stroke, large vessel occlusion, protocol

## Abstract

**Background:** Stent retriever thrombectomy is the standard treatment for acute ischemic stroke (AIS) with large vessel occlusion (LVO) in anterior circulation. The aim of the trial is to evaluate whether the new thrombectomy device-Skyflow can achieve the same safety and efficacy as Solitaire FR in the treatment.

**Method:** This study is a prospective, multicenter, stratified randomized, single blind, paralleled, positive controlled, non-inferiority clinical trial. The safety and efficacy of vascular recanalization in AIS patients who are treated with either a new thrombectomy device-Skyflow or with Solitaire FR and within 8 h of symptom onset will be compared. A total of 192 patients will be enrolled, each group with 96 patients. The primary endpoint is successful recanalization rate after the operation. The secondary efficacy endpoints are the time from artery puncture to successful recanalization (mTICI 2b-3), NIHSS scores of 24 h (18–36 h), and 7 ± 2 days after the operation, mRS scores, and the rate of patients with mRS 0–2 scores 90 ± 14 days after the operation, and the success rate of instrument operation. The safety endpoints are the rate of symptomatic intracranial hemorrhage (sICH) and subarachnoid hemorrhage at 24 h (18–36 h) post-operation, incidence of adverse events (AE) and serious adverse events (SAE), all-cause mortality, and incidence of device defects.

**Discussion:** This trial will provide information on the safety and efficacy of Sky-flow stent retriever in the treatment of AIS patients with anterior circulation LVO. The success of this trial will be the basis for the product to be finally officially listed and applied in China.

**Trial registration:** Registered on 11 March 2018 with Chinese clinical trial registry. Registration number is ChiCTR1800015166.

## Introduction

Stroke is one of the main causes of human disability and death. Acute ischemic stroke (AIS) accounts for about 80% of all types of stroke (1). The key to AIS treatment is to recanalize the occluded blood vessel as soon as possible and salvage the ischemic penumbra. Endovascular treatment (EVT) in patients with large vessel occlusion (LVO) has attracted much attention because of many advantages such as rapid recanalization, lower hemorrhagic transformation rate, and extended stroke interventional therapy time window (2–5).

The invention of the stent retriever is a huge advancement of EVT, which has been reported to achieve a higher recanalization and favorable clinical outcome rate (2). The stent retriever uses a temporary stent to capture the thrombus, and restore blood flow by squeezing the peripheral blood vessel wall and moving the thrombus. When the stent is withdrawn, the thrombus is captured in the stent gap and removed together with the stent. The food and drug administration (FDA) of USA approved Solitaire (Medtronic/ev3) and Trevo (Strike) stent retriever to be mainly used for the treatment of large vessel occlusive stroke in 2012. The Solitaire FR With the Intention for Thrombectomy (SWIFT) study (6) and Thrombectomy Revascularization of Large Vessel Occlusions in Acute Ischemic Stroke (TREVO 2) trial (7) declared that MT with the Solitaire FR stent (ev3 Covidien) and the Trevo stent (Stryker Neurovascular) achieved better revascularization than those with the Merci retriever device. And it is recommended to use the stent retriever for acute mechanical thrombectomy (MT) (Class I, Level A) in the treatment of acute anterior circulation LVO stroke in guidelines for the early management of patients with AIS (8).

At present, the China Food and Drug Administration (CFDA) has approved several thrombectomy devices of foreign companies to be listed domestically, including Solitaire FR (Medtronic/ev3), Trevo thrombectomy system (Stryker), and Revive self-expanding intracranial thrombectomy device (Codman). In recent studies, the new mechanical thrombectomy devices, such as EmboTrap, Tigertriever and NeVa^TM^, have also achieved a high recanalization rate and good clinical outcome (9–12). The safety and efficacy of the domestically-made mechanical thrombectomy devices, such as Tonbridge stent retriever, JRecan stent retriever, and Reco stent retriever, have been verified in animal experiments or clinical trials, but they have not yet been widely used (13–15). In China, stent retrievers are still mainly imported and are expensive, making a lot of patients unable to receive effective treatment.

**Figure 1:**
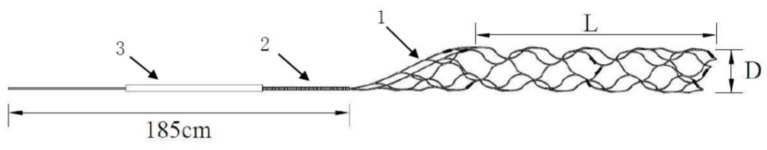
Description of stent composition: there are different sizes of stent retriever, including TD 320, TD420, TD430, and TD440 requiring a compatible microcatheter of 0.021 inch and TD620 and TD630 requiring a compatible microcatheter of 0.027 inch. Stent retriever with double helix structure, partial large mesh and full development design (1), pushing wire 185 cm with a variable-diameter structure coiled with platinum alloy coil in the distal (2), introducer sheath (3), diameter of stent, 3 mm for TD 320, 4 mm for TD420, TD430, and TD440, 6 mm for TD620 and TD630 (D), and stent retriever effective length, 20 mm for TD 320, TD420, and TD620, 30 mm for TD430 and TD630, and 40 mm for TD440 (L).

In order to better meet the domestic AIS patients and the ever-increasing clinical needs, Skynor medical (Shanghai) co. LTD designed a new thrombectomy device-Skyflow which has been approved by CFDA. The thrombectomy device is mainly composed of stent retriever and an introducer sheath, and the stent retriever including a stent and a pushing wire. There are marks of platinum alloy on the stent, which can be seen through DSA or X-ray. The stent can be released and retrieved by pushing and pulling the delivery wire. And the stent retriever is compressed into an introducer sheath, which is designed to facilitate stent loading into the microcatheter. The length of the delivery wire of the device is 185 cm, and the inner diameter of the compatible micro catheter is 0.021″/0.027″. It is divided into different specifications according to stent diameter of 3, 4, or 6 mm (Figure 1).

This clinical trial aims to evaluate the safety and efficacy of the new thrombectomy device-Skyflow for patients of AIS with large vessel occlusion in anterior, and provide a basis for the product to be finally officially listed and applied.

## Method

### Objective

The aim of the trial is to evaluate the safety and efficacy of the new thrombectomy device-Skyflow in the treatment of AIS to provide a basis for product registration, listing, and application.

### Study Design

This study is a prospective, multicenter, stratified randomized, single blind, paralleled, positive controlled, non-inferiority clinical trial comparing the safety and efficacy of recanalizing occluded vessel for patients with AIS within 8 h of symptom onset treated with either a new thrombectomy device-Skyflow (TD 320, TD420, TD430, TD440, TD620, or TD630) or with Solitaire FR (SFR-4-15, SFR-4-20, SFR-6-20, or SFR-6-30). The study will be conducted in more than 10 clinical trial centers. A total of 192 patients who fulfill the inclusion and exclusion criteria and provide informed consent will be randomized into either a treatment group or a control group in the ratio of one-to-one. The treatment group will receive mechanical thrombectomy with the properly sized new device-Skyflow and the control group with Solitaire FR to restore blood flow of the occluded vessel and improve clinical symptoms. Subjects will be followed up for 5 times after enrollment, respectively, before operation, intraoperation, 24 h after operation, 7 days after operation, and 90 days after operation. If there is an adverse event during the clinical trial, the investigator needs to observe outcome of the adverse event. All assessment indexes were recorded to evaluate the safety and efficacy of the experimental thrombectomy device in the treatment of AIS. Table 1 is a brief summary of visits and assessment schedule.

**Table 1:** Visits and assessment schedule.

	**Visit 1**	**Visit 2**	**Visit 3**	**Visit 4**	**Visit 5**
	**−1~0 day before operation**	**During operation**	**24 h after operation (18–36 h)**	**7** **±** **2 day after operation**	**7** **±** **2 day after operation**
Informed consent	×				
Inclusion/exclusion criteria	×	×			
Random allocation		×			
Demographic data	×				
History of current illness.	×				
Past medical history	×				
Vital signs	×			×	
NIHSS score	×		×	×	
mRS score	×				×
Blood routine test^1^	×			×	
Routine coagulation test^2^	×			×	
Liver function test^3^				×	
Renal function test^4^	×			×	
Random blood glucose	×				
Pregnancy test^5^	×				
12-lead electrocardiogram	×			×	
Brain CT or MR^6^	×		×		
Cerebrovascular DSA^7^		×			
The use of surgical and experimental instruments		×			
Concomitant medication^8^	×	×	×	×	×
Adverse events		×	×	×	×
Device defects		×			

1 *blood routine test: Hgb, Hemoglobin Content; RBC, Red Blood Cells Count; WBC, White Blood Cell Count; NEUT, Absolute Neutrophil Count; NEUT%, neutrophil ratio; PLT, Platelet Count*;

2 *routine coagulation test: PT, Prothrombin Time; APTT, Activated Partial Thromboplastin Time; INR, the international normalized ratio*;

3 *liver function test: AST, aspartate transaminase; ALT, alanine transaminase; STB, serum total bilirubin; TP, Serum Total Protein; ALB, serum albumin*;

4 *renal function test: Scr, serum creatinine; BUN, Blood Urea Nitrogen*;

5 *pregnancy test: blood HCG or urine pregnant test, Suitable for women of childbearing age*;

6 *brain CT or MR: identify whether there is intracranial hemorrhage, massive cerebral infarction or angiemphraxis before the interventional procedure, and whether there is intracranial hemorrhage after procedure*;

7 *cerebrovascular DSA: identify whether there is initial carotid artery occlusion, carotid artery dissection or arteritis, whether there is a tortuous vessel path that makes it difficult for the test/control device to reach the target position, the target vessel and the diseased segment, the proximal diameter of the occluded vessel, mTICI before, and after the operation*;

8 *record the use of anticoagulant, antiplatelet drugs, and drug for adverse events*.

### Participants

#### Inclusion Criteria

Patients eligible for inclusion will meet the following criteria (6, 16, 17):

1. Aged 18–80 years, clinical signs consistent with AIS.
2. Clinical signs consistent with AIS; Subjects who are able to be treated within 8 h of onset of stroke symptoms; NIHSS ≥ 6 and <30 at the time of randomization.
3. Occlusion in the intracranial internal carotid, or M1/M2 segment of the middle cerebral artery (MCA) confirmed by DSA.
4. Prestroke Modified Rankin Score ≤ 2.
5. Informed consent of the patient or family member.

#### Exclusion Criteria

Patients will be excluded if they meet any of the following criteria:

1. CT or MRI evidence of hemorrhage or infarction involving greater than 1/3 of the MCA territory (or in other territories, > 70 ml of tissue on presentation, or CT/DWI-ASPECT <6).
2. Bilateral internal carotid artery occlusion detected by DSA.
3. Initial segment of internal carotid artery occlusion or carotid artery dissection detected by DSA.
4. The route artery is too tortuous for test/control equipment to reach the target position.
5. Severe and sustained hypertension (defined as systolic blood pressure >185 mmHg or diastolic blood pressure >110 mm Hg under the treatment of antihypertensive drug).
6. Baseline platelet count <40 ×10^*^9/L or recent oral anticoagulant therapy with INR>3.
7. Baseline blood glucose <2.8 mmol/L or > 22.20 mmol/L.
8. Having a heart, lung, liver, and kidney function failure or other serious diseases, such as intracranial malignant tumors, intracranial arteriovenous malformation, arteritis, systemic infection, and activity of disseminated intravascular coagulation, with serious disabling stroke within 6 months and severe mental illness.
9. Can't cooperate with interventional surgery or can't tolerate.
10. Life expectancy of less than 90 days.
11. Known serious sensitivity to radiographic contrast agents.
12. Female who is pregnant or lactating or has a positive pregnancy test at time of admission.
13. Involved in other drugs or equipment trials within 3 months before randomization.

### Criteria and Procedures for Discontinuing Trial/Trial Treatment

#### Dropout Criteria

All subjects who sign an informed consent form and are selected to be enrolled in the central randomization system are entitled to withdraw from the clinical trial at any time. As long as they didn't complete the clinical trial observation, it should be considered as dropout cases.

#### Management of Dropout Cases

When a subject drops out, the researcher must fill in the reason in the CRF, and contact the subject as much as possible to complete the assessment items and record the last follow-up time. The CRF should be reserved for future reference. For cases who drop out due to adverse events, in addition to being recorded in the CRF, they should be also included in the adverse event evaluation. The random number of dropout cases cannot be replaced.

#### Elimination Criteria

Subjects who meet one of the following criteria should be eliminated: being falsely enrolled or neither using test devices nor control devices.

#### Management of Eliminated Cases

The researcher must fill in the elimination reason in the CRF and the CRF should be reserved for future reference. The random number of eliminated cases cannot be replaced.

#### Full Termination Criteria

The experiment should be terminated due to any of the following reasons: there are serious safety issues with the experimental device; the experimental device has a poor effect and there is no need to continue the test; there are major mistakes in the protocol; and there are funding or management problems of sponsor.

### Treatment and Intervention

For all the enrolled patients, stent retriever thrombectomy is the first-line approach. Skyflow device is for the trial group and solitaire FR for control group. Patients who are eligible for intravenous thrombolysis according to the guidelines were given 0.9 mg/kg alteplase. General or local anesthesia was chosen based on the patients' clinical condition and heparin was used selectively. Stent retriever thrombectomy can be performed repeatedly and generally no less than 3 times before rescue therapy. If the intracranial occluded blood vessel had significant stenosis or other lesions after mechanical thrombectomy with stent retriever, the researcher should evaluate whether to perform the aspiration thrombectomy, balloon dilatation, stent implantation or other rescue therapy based on the subject's clinical conditions. If adjuvant rescue treatment was performed, DSA should be recorded and mTICI classification should be reevaluated. Follow-up examinations and visits were carried out according to the test procedure.

### Management of Concomitant Medication

Drugs and treatments for underlying diseases, comorbidities or adverse events are permitted. But devices with similar effects to the experimental device or the control device are prohibited for all subjects during clinical trials. If the subject used other drugs and treatments for various reasons during the study period, the investigator needed to record this case in details in the original medical record for future reference and evaluate the possible bias in clinical trial results.

### Endpoint Measurement

The primary endpoint is successful recanalization rate. It is defined as the proportion of target vessels achieving successful recanalization [modified Thrombolysis in Cerebral Infarction (mTICI) 2b-3].

The secondary efficacy endpoints are the times from artery puncture to successful recanalization (mTICI 2b-3), NIHSS scores 7 ± 2days after the operation, mRS scores and the rate of patients with 0–2 scores 90 ± 14 days after the operation, and the success rate of instrument operation (the rate of instruments being successfully delivered, released, and withdrawn in this group).

The safety endpoints are the rate of symptomatic intracranial hemorrhage (sICH) and subarachnoid hemorrhage at 24 h (18–36 h) post-operation, incidence of adverse events (AE) and serious adverse events (SAE), all-cause mortality and device defects.

sICH is defined as any ICH associated with neurological deterioration assessed by 4 or more points increase on the NIHSS score within 18–36 h. Adverse events are unfavorable medical events that occur during clinical trials, whether related to the test device or not, including hematoma or hemorrhage at the site of puncture, air embolism, infection, distal embolization, vascular spasm, thrombosis, vascular dissection and perforation, embolism, acute occlusion, ischemia, intracranial hemorrhage, pseudoaneurysm formation, neurological deficits including stroke, and death, and device deformation, fracture and malfunction. Serious adverse events are defined as any events which can cause deaths or serious health deterioration that occur during clinical trials, including fatal diseases or injuries, permanent defects of body structure or function, events that need hospitalization or can prolonged hospitalization, events that need medical or surgical intervention in order to avoid permanent defects in body structure or function, and events that can cause fetal distress, fetal death, or congenital anomalies and congenital defects. Device defects refer to the unreasonable risks of medical devices under normal use that may endanger human health and life safety during the clinical trials, such as incorrect identification and device breakage.

### Management of Adverse Events

All adverse events that occurred during the trial must be faithfully recorded. Researchers should give targeted treatment and follow-up for adverse events until the symptoms disappear or become stable.

For serious adverse events in the clinical trial, the investigator should immediately take appropriate treatment measures to the subject, and at the same time report to the medical device clinical trial management department in writing and notify the sponsor in writing through the department. The medical device clinical trial management department should report to the relevant ethics committee, the Food and Drug Administration and the health and family planning department of the province, autonomous region, and municipality in writing within 24 h. For deaths, clinical trial institutions and researchers should provide all necessary information to the ethics committee and sponsors.

When device defect occurs, replace it with the same device or take conventional treatment. For device defects that cause or may cause serious adverse events, the sponsor should report to the registered FDA and the health and family planning authority at the same level within 5 working days. At the same time, it should notify other clinical trial institutions and researchers participating in the trial and timely informs the ethics committee of the clinical trial institution through the medical device clinical trials management department.

### Randomization

Immediately after DSA and prior to mechanical thrombectomy, 192 patients are randomly allocated in a one-to-one ratio to either experimental or control group using an Internet-based Central Random System (IWRS). When grouping the patients, stratification will be performed to control key indicators by the clinical trial center and the baseline NIHSS score (divided by NIHSS score ≤ 17 points or > 17 points) (2).

### Reduction and Avoidance of Bias

The following measures have been taken to reduce and avoid bias:

NIHSS and mRS score were assessed by physician independent of the operator in each clinical trial center. The images are reviewed by the core laboratory to assess time of successful recanalization and the grade of the blood flow of the target vessel before and after the operation. When the result is different from the clinician's judgment, the core laboratory result shall prevail.

Sponsors, clinical trial supervisors, and researchers took corresponding measures to reduce the bias on results during the trial, including training for investigators before the start of the study to make sure they are familiar with research procedure and device operation, selecting skilled and experienced surgeons who has completed more than 30 mechanical thrombectomy operations, ensuring that the experimental protocol is strictly followed, following the requirements of relevant treatment guidelines, and data verification after the completion of clinical trials.

### Monitoring Plan

The sponsor should select qualified supervisors to perform supervisory duties on all participating institutions. Before the trial, supervisors should confirm the clinical trial institution has the appropriate conditions including the staffing and training meeting the requirements, well-equipped laboratory, good working condition, sufficient number of subjects, and the researchers are familiar with the test requirements. They should also supervise whether the clinical trial institutions and researchers comply with relevant regulations, and supervise medical device samples and related equipment during the trial. And supervisors must confirm that each subject signs an informed consent before participating in the clinical trial and all case report forms are filled correctly and consistent with the original data, and ensure that all clinical trial related documents received by investigators are new versions. Detailed information must be recorded for patients who withdrew from the experiment, don't follow the informed consent form, and are not followed up completely, as well as all adverse events, complications, and other device defects.

### Ethical Considerations and Informed Consent

This clinical trial comply with the Declaration of Helsinki, Medical Device Clinical Trial Quality Management Regulations and Chinese relevant laws and regulations. Before the start of the trial, the protocol must be approved by the ethics committee of each research center. The investigators should provide the subjects or their guardians complete information about the trial and should not compel or induce subjects to participate in the trial in improper ways. If the subjects have a disturbance of consciousness or have a difficulty in understanding language, the investigator should fully explain the details of the clinical trial to their guardian, and the guardian and investigators shall sign their name and date on the written informed consent prior to enrollment and keep it in the research files.

### Sample Size

The calculation of the sample size is based on the successful recanalization rate evaluated during the operation. Based on the results of the North American Solitaire post-marketing study (18), the meta-analysis of thrombectomy devices (2), and a multi-center registered clinical trial (EAST) led by Professor Miao Zhongrong of Tiantan Hospital, it is expected that the test group can reach the same efficiency when the sponsor and clinicians assumed the rate of successful recanalization after treatment was about 90% in the control group. Eventually, a total of 192 patients were enrolled according to calculation based on statistical principles, 96 cases in each group with a 12.5% non-inferiority margin, a 5% significance level (two-tailed), an 80% statistical power and a 5% dropout rate.

### Statistical Analysis

For descriptive analysis, enumeration data will be expressed by frequency and composition ratio, and the measurement data will be expressed by the mean, standard deviation, maximum, minimum, median, 25th and 75th quantile.

For baseline demographics, all date will be analyzed based on full analysis set. Continuous calibration Chi-square tests or Fisher's exact tests will be used for comparison of enumeration data between groups. Group *t-*test will be used for comparison of normally distributed measurement data between groups, and Wilcoxon Rank Sum test for non-normally distributed measurement data.

For the primary outcome, the successful recanalization rate of blood vessels, the CMH chi-square analysis with adjusted center effect will be used for comparison between groups based on full analysis set and per protocol set. In addition to the success rate, the difference in the success rate between the experimental group and the control group and the 95% confidence interval with the lower bound greater than −12.5% will be also estimated.

Other efficacy indicators comparation between groups will be the same as the baseline analysis. The paired *t-*test will be used for the intra-group comparison of normally distributed measurement data and the Wilcoxon Sign Rank test for the intra-group comparison of non-normally distributed measurement data.

For safety evaluation, the number and proportion will be used to describe cases that were normal before treatment and abnormal after treatment in the full analysis set. Adverse events will be described by number and incidence, and this incidence will be tested by continuous calibration Chi-square tests or Fisher's exact tests. At the same time, the specific manifestations and extent of all adverse events in each group and their relationship with the research products will be described in detail.

For missing data, only the missing primary endpoint indicators will be carried forward during analysis, while other indicators will be not. Incorrect and unreasonable data will be checked by the electronic data capture (Electronic Data Capture, EDC) system and the question will be answered by the researcher before statistical analysis. For patients withdrawing from the trial, the information will still be included in the final statistical analysis.

All data will be analyzed using SAS9.4 software and all statistical analysis will be performed at the two-sided 0.05 significance level (except for special instructions).

### Date Management

The data management will be in the charge of the Medical Statistics Department of the National Cardiovascular Disease Center, and the data collection will be performed by them using EDC system. Researchers are responsible for the quality of the data entry, and should ensure the authenticity and completeness of the data. Data verification is completed by both the EDC system and the data administrator. The modification and locking of data need to be approved by the sponsor, the person in charge of data management, and the person in charge of statistics.

## Discussion

Stent retriever has recently been widely used for thrombectomy, and has been reported to be associated with faster reperfusion and higher rate of successful recanalization in endovascular treatment of intracranial LVO (19). However, the research and development of domestic stent retrievers is relatively slow and few devices have been verified by clinical trials (15), which greatly hinders the progress of endovascular treatment in China. The purpose of this trial is to verify whether the new thrombectomy device-Skyflow has similar safety and efficacy compared with Solitaire FR in the treatment of acute LVO.

Compared with other stent retriever, the device-Skyflow has some unique structural features (15, 20, 21). The double helix structure makes stent have excellent flexibility and adhesion, which improve the thrombus removal rate, while avoid excessive damage for intracranial blood vessels. Partial large mesh design and the wall thickness greater than the rod width can effectively embed and remove the thrombus. Full development design helps the doctor to determine the position and length of the stent and its unfolding shape which can improve the success rate of the operation. The pushing wire of the thrombectomy device is made of nickel-titanium material, and the distal end is designed as a variable-diameter structure coiled with platinum alloy coil, which improves the pushability and visualization of the product. And the device has different models and specifications, which can meet the requirements of occluded vessel with different diameters. All these features provide theoretical basis for the safety and efficacy of the test product. But relative to the reel overlap design and multi-target combination with thrombus of solitaire FR, the Skyflow has a weaker cutting and grasping ability for thrombus. In addition, compared to solitaire FR, the delivering catheter of Skyflow device is larger, which may make it difficult to pass the Atherosclerotic stenosis.

The Solitaire FR is a representative of stent retriever and the technology is mature. The main structure, action principle, treatment method, indications, etc. are similar to the test product (20). Therefore, it is reasonable to choose Solitaire FR device as a control product in our trial. In addition, this trial has strict inclusion and exclusion criteria. DSA is used to identify the occlusion site, and CT or MRI to exclude patients with preoperative intracranial hemorrhage and large core infarct. And the trial adopted a stratified random approach to avoid the influence on the results of preoperative NIHSS score and different research centers. A series of other measures have also been taken to ensure the quality of research, including the supervision of the experimental equipment of clinical trial institutions and relevant training for the investigators, quality control and monitoring, and data management and check after the research etc. These can make the trial results more convincing. But this is a single-blind clinical trial. The researchers cannot be blinded due to the difference of appearance between the test device and the control device, which can cause an impact on the trial results.

In conclusion, results of this trial will provide information on the safety and efficacy of Skyflow stent retriever. The success of this trial will provide a new option for the treatment for patients of AIS with LVO in China.

## Ethics Statement

The studies involving human participants were reviewed and approved by Ethics Committee of Drugs (Devices) Clinical Experiment in Henan provincial People's Hospital and other research centers participating in the clinical trial. The patients/participants provided their written informed consent to participate in this study.

## Author Contributions

TL and YH conceived of the study. ZL and LZ designed the study. HL contributed to the draft of the manuscript. TZ, QW, YH, and XS contributed to the revision of the manuscript. All authors read and approved the final manuscript.

## Conflict of Interest

The authors declare that the research was conducted in the absence of any commercial or financial relationships that could be construed as a potential conflict of interest.
